# Retroperitoneal totally endoscopic prosthetic repair of lumbar hernia

**DOI:** 10.1038/s41598-023-48226-x

**Published:** 2023-11-25

**Authors:** Haoran Li, Zhengwu Cheng, Wenwu Yan, Xunzi Hu, Junfeng Wang

**Affiliations:** Department of Gastrointestinal Surgery, The First Affiliated Hospital of Wannan Medical College, 2 Zheshan West Road, Wuhu, 241000 Anhui Province China

**Keywords:** Anatomy, Diseases, Medical research

## Abstract

Lumbar hernia is a rare lateral abdominal wall hernia. Various surgical repair strategies have been recorded, but there is currently no unified standard. A Chinese surgeon recently revealed a novel technique for treating primary lumbar hernia called retroperitoneal totally endoscopic prosthetic repair (R-TEP). We have made a further exploration of this method and successfully used it in the treatment of secondary lumbar hernia. We successfully performed R-TEP on three patients with lumbar hernias. All patients were female with an average age of 64 years (51–71 years). Two patients each had a primary upper lumbar hernia, while one patient had a secondary lumbar hernia. With a mean operative time of 77 min (60–105 min), all operations were performed successfully. The average visual analogue scale (VAS) was 1.3 points (1–2 points) on the second day following surgery. The mean postoperative hospital stay was 2.3 days (2–3 days). No postoperative complications occurred. During a mean follow-up period of 19 months (10–24 months), there was no recurrence of the hernia, chronic pain or mesh infection. Therefore, R-TEP is safe and effective for both primary and secondary lumbar hernia. Anti-adhesive coated meshes are not required, making this a cost-effective procedure that is worthy of recommendation.

## Introduction

Lumbar hernia refers to an external abdominal hernia that occurs in the posterior lateral wall of the abdomen between the 12th rib and the iliac crest, where retroperitoneal fat and/or abdominal tissues and organs protruded into the body surface through the weakness of the upper lumbar triangle or the lower lumbar triangle^1^. Although abdominal wall hernia has a common incidence of 4–5% worldwide, the lumbar hernia is rare^2,3^. According to Hafner, a surgeon only has one opportunity in a lifetime to diagnose and surgically treat a lumbar hernia^2^. Lumbar hernia is classified as congenital and acquired according to its causes. Among them, congenital lumbar hernia accounts for 20%, which is primarily brought on by dysplasia of the lumbar dorsal muscles or fascia during the embryonic stage^4^. Adult lumbar hernia can be divided into primary and secondary types.The primary lumbar hernia may be associated with muscle atrophy and increased intra-abdominal pressure and is more common in the elderly. Secondary lumbar hernia is mostly caused by external factors such as lumbar dorsal surgery, trauma, infection and abdominal wall nerve injury. Statistics show that primary lumbar hernias are more common, making for about 55% of all lumbar hernias^5^. According to different locations, the primary lumbar hernia can be separated into upper lumbar triangle hernia (Grynfeltt-Lesshaft hernia) and lower lumbar triangle hernia (Petit hernia)^6^.

Surgery is the main treatment for adult lumbar hernias if the diagnosis is established. The traditional Dowd operation, which involves high ligating the hernia sac, repairing the transversalis fascia and the muscle fascia surrounding the lumbar triangle, and imbricating overlapping suture, is one surgical technique^7^. However, due to the high local tension and subsequent postoperative obvious pain, it has been rarely carried out. At present, mesh repair has become the primary surgical procedure for a lumbar hernia. According to Pascal's hydrostatic theory, intra-abdominal pressure is evenly distributed throughout the mesh, allowing it to adhere and fix to the abdominal wall with a firm repair and satisfactory outcomes^8,9^. In the past, open Sublay surgery was mainly performed^10^. With the development of laparoscopic technology, intraperitoneal onlay mesh (IPOM)^11^, transabdominal preperitoneal repair (TAPP)^12^ and transabdominal partially extraperitoneal repair (TAPE)^13^are frequently employed in clinical practice.

In the early twenty-first century, Meink^14^ and Habib^15^each made an attempt to perform retroperitoneal totally endoscopic prosthetic repair (R-TEP) for lumbar hernia patients. There have been few reports since then. In recent years, Li^1^ has effectively treated 10 cases of primary lumbar hernia using R-TEP. He believed that, with proficiency in anatomy, this technique was safe, feasible, and effective in treating lumbar hernia. More importantly, R-TEP should be a more reasonable procedure, avoiding the shortcomings of previous procedures. He has made useful further attempts to treat lumbar hernia, but the number of reported cases was small, the follow-up time was short, and there was a lack of patients with secondary lumbar hernia.

We have accumulated a lot of experience with TEP for the treatment of inguinal hernias. On this basis, R-TEP was recently performed successfully for 2 patients with primary upper lumbar hernias and 1 patient with a secondary upper lumbar hernia, and all patients’ prognoses were good. The following report aims to further explore the clinical value of this operation in the treatment of lumbar hernia.

## Material and methods

**Figure 1:**
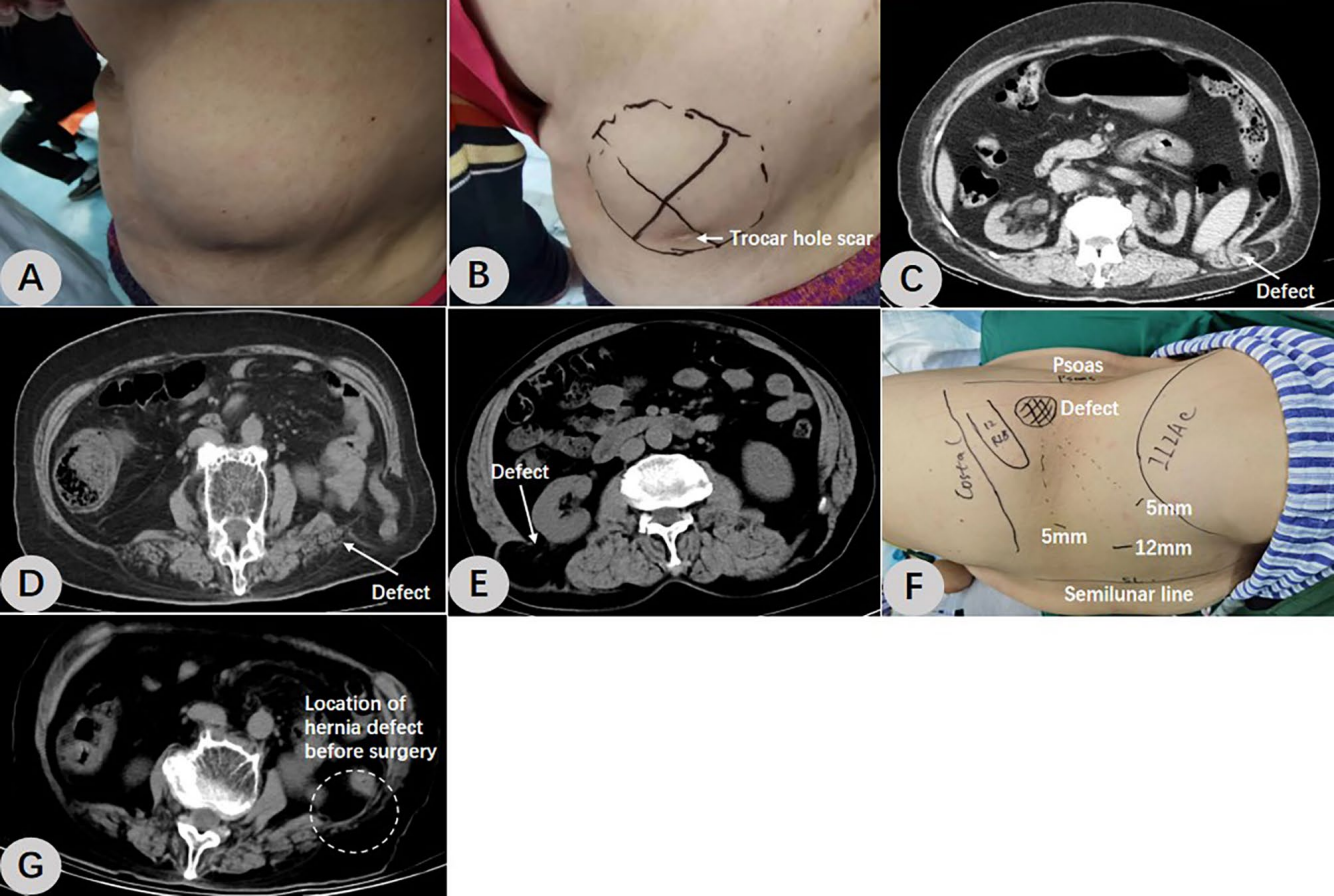
Patients’ signs, CT images, and preoperative surgical markers. (**A**) A patient with a primary left-side Grynfeltt hernia. (**B**) A patient with a secondary left-side lumbar hernia. (**C**) CT revealed a left-side Grynfeltt hernia. (**D**) CT revealed a secondary left-side lumbar hernia. (**E**) CT revealed a right-side Grynfeltt hernia. (**F**) The patient was positioned in the healthy lateral decubitus position with waist hyperextension. (**G**) The follow-up CT image of the second patient.

This retrospective study was conducted in the First Affiliated Hospital of Wannan Medical College in China. Three consecutive adults with lumbar hernias underwent retroperitoneal TEP repair from March 2021 to May 2022. Among them, two had a primary upper lumbar hernia (Fig. 1A) and one had a secondary lumbar hernia (Fig. 1B). All operations were performed by the same surgeon. Data including patients’ demographics and diagnoses were collected retrospectively (Table 1). The main endpoints of the study were hernia recurrence, intraoperative and postoperative complications, and postoperative pain. Complications including hematomas, infection, seromas, and any serious adverse events were recorded and analyzed.

**Table 1:** Patients’ general demographic and diagnoses data.

Patient	Age (years)	Gender	BMI (kg/m^2^)	ASA	Types of hernia	Side
1	70	Female	29.05	I	Primary	Left
2	71	Female	25.39	II	Secondary	Left
3	51	Female	28.40	I	Primary	Right

*BMI* body mass index, *ASA* American Society of Anesthesiologists.

This retrospective study involving human participants was in accordance with the ethical standards of the institutional and national research committee and with the 1964 Helsinki Declaration and its later amendments or comparable ethical standards. The study was approved by the Independent Ethics Committee (IEC) of the First Affiliated Hospital of Wannan Medical College (IRB number 202316). All the patients provided their written informed consent for participation in the study and for the publication of their data and photographs.

**Patient 1** A 70-year-old woman was admitted to the hospital on March 24, 2021, due to "a reversible mass on the left waist for two months". Half of the spherical mass could be seen on the left waist when the patient was standing or on exertion, and the mass disappeared spontaneously in the right-lying position. The patient was healthy in the past and denied a history of hypertension, diabetes, coronary heart disease, surgery, or trauma. The preoperative American Society of Anesthesiologists (ASA) rating was grade I. The patient's height was 153 cm, weight 68 kg, and body mass index (BMI) 29.05 kg/m2. The size of the hernia sac was approximately 5 cm × 4 cm, and an abdominal CT scan revealed a fascia defect with a diameter of about 3 cm at the left superior lumbar triangle. The bowel herniated out of the abdomen, and a primary left superior lumbar hernia was diagnosed (Fig. 1C).

**Patient 2** A 71-year-old woman was admitted to the hospital on April 5, 2021, due to "a reversible mass on the left waist for one month". When the patient was standing or exerting herself, half of the spherical mass could be seen on the left waist, and the mass spontaneously vanished in the right-lying position (Fig. 1B). The patient had a more than 10-year history of hypertension, and oral amlodipine was effective in lowering her blood pressure. She denied having diabetes or coronary heart disease and underwent “left renal cyst resection” in 2018. Grade II was her preoperative ASA rating. The patient had a height of 160 cm, a weight of 65 kg, and a BMI of 25.39 kg/m2. The size of the hernia sac was approximately 5 cm × 4 cm, and an abdominal CT scan revealed a fascia defect with a diameter of about 3 cm at the left superior lumbar triangle. The bowel and a little mesentery herniated out of the abdominal cavity, and a secondary left lumbar hernia was diagnosed (Fig. 1D).

**Patient 3** A 51-year-old woman was admitted to the hospital on May 6, 2022, due to "a reversible mass on the right waist for half a year". Half of the spherical mass could be seen on the right waist when the patient was standing or on exertion, and the mass disappeared spontaneously in the left-lying position. She denied having ever experienced trauma, surgery, hypertension, diabetes, or coronary heart disease and stated that she had previously been healthy. Her preoperative ASA rating was grade I. She was 157 cm tall, weighed 70 kg, and had a BMI of 28.40 kg/m2. The size of the hernia sac was about 5 cm × 4 cm, and an abdominal CT scan showed a facia defect at the right superior lumbar triangle that was approximately 3 cm in diameter. Retroperitoneal fat was seen on the right posterior lateral wall of the abdomen, and a primary right superior lumbar hernia was diagnosed (Fig. 1E).

## Surgical technique

The preoperative evaluation including laboratory examination, electrocardiogram, echocardiography, abdominal CT scan, chest HRCT, etc., was perfect, and the diagnosis of lumbar hernia was clear. There was no obvious contraindication for surgery and the underlying diseases were under good control. All the 3 patients were planned to receive R-TEP. Due to the adhesion of the surgical area and the complex anatomy, the surgical approach may be altered to IPOM or TAPP during the operation. Before surgery, patients and their families accepted the aforementioned surgical methods and signed informed consent.

After tracheal intubation and general anesthesia were stable, the patients were placed in the healthy lateral decubitus position with waist hyperextension, and the surgical regions were disinfected and covered with towels. Laparoscopic monitor screens were positioned on the affected side of the patients, while the surgeon stood on the healthy side.

**Figure 2:**
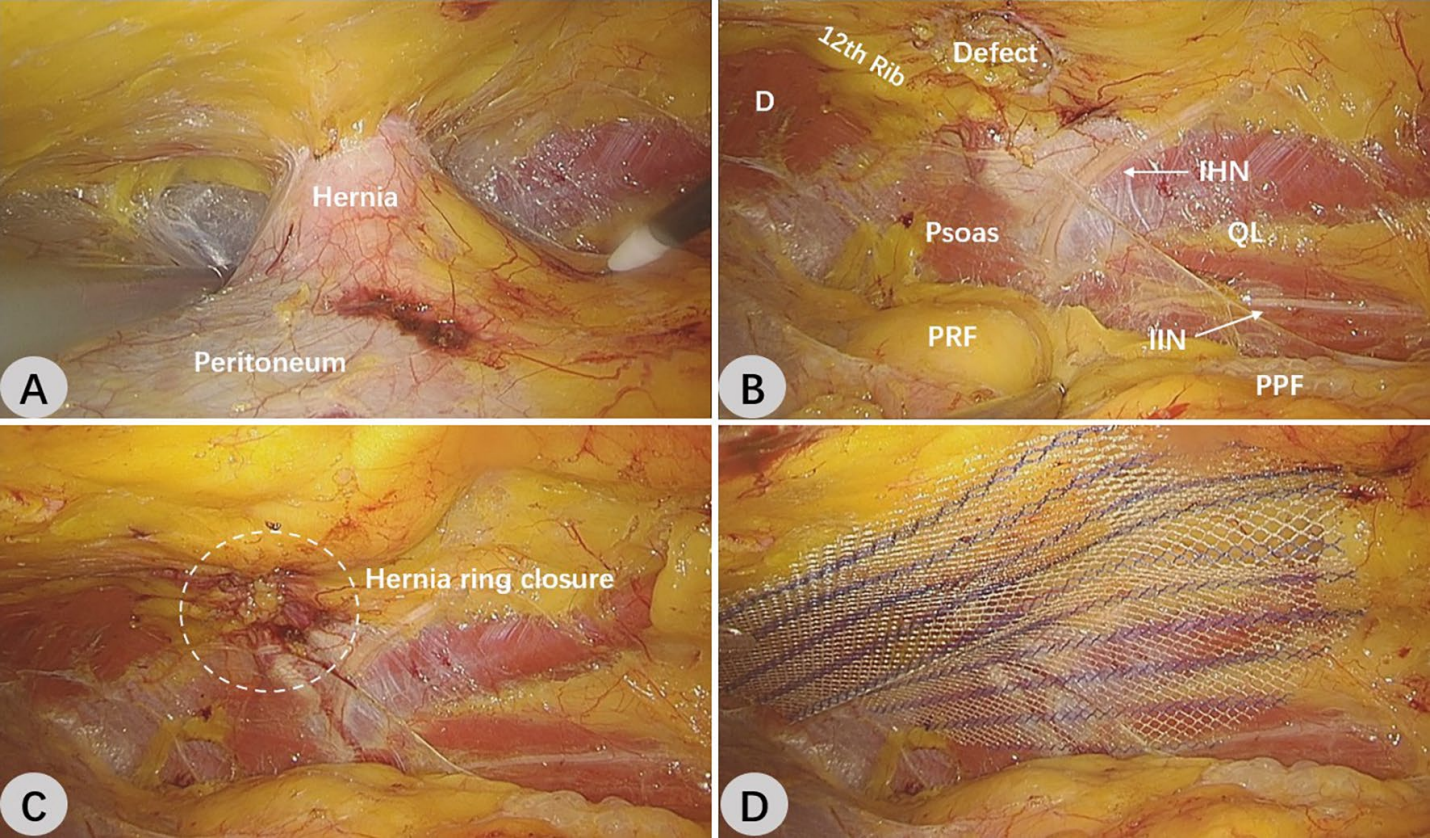
Intraoperative images of R-TEP in the treatment of primary lumbar hernia. (**A**) The hernia sac was seen to protrude through the superior lumbar triangle under retroperitoneal endoscopic vision. (**B**) The dissected retroperitoneal space. Note the courses of the nerves. (**C**) Closure of the hernia ring. (**D**) A partially absorbable polypropylene mesh was placed to cover the hernia defect without traumatic fixation. *D* diaphragm, *PRF* perirenal fat, *IHN* iliohypogastric nerve, *QL* quadratus lumborum, *IIN* ilioinguinal nerve, *PPF* preperitoneal fat.

1. *Establishment of retroperitoneal space* Three trocars were inserted during the operation. The establishment of the observation hole is the first and also crucial step. A 1.2 cm long skin incision was made in the anterior axillary line on the affected side, about 2 cm from the upper edge of the anterior superior iliac spine. Two separating forceps were used to bluntly separate the subcutaneous fat in order to view the aponeurosis of the external abdominal oblique. The aponeurosis was lifted and cut about 1 cm in length. The extraperitoneal fat was exposed by bluntly pulling the abdominal internal oblique and transverse abdominal muscle fibers to the sides using two thyroid retractors. A blunt fan-shaped separation was made using the fingers in the extraperitoneal space, and then a 12 mm trocar was inserted. After the pneumoperitoneum was connected and intra-abdominal pressure was set at 13 mmHg (1 mmHg = 0.133 kPa), the endoscope was extended. The extraperitoneal space was further separated superiorly and laterally using the endoscope-push method. Under direct vision, a 5 mm trocar was inserted approximately 3 cm below the lower edge of the costal arch in the anterior axillary line and about 2 cm above the upper edge of the iliac crest in the mid-axillary line. (Fig. 1F)
2. *Retroperitoneal space expansion* The extraperitoneal space was further expanded and separated. Forward to the anterior axillary line; Laterally to the lateral psoas major muscle; Ascending at least 3 cm above the 12th rib; Descending to 3 cm below the inner edge of the iliac crest. During the separation, the hernia sac was encountered and it needed to be completely dissociated. A portion of the lumbar hernia lacks a hernia sac and is filled with extraperitoneal adipose tissue, which can be directly pulled back. (Fig. 2A, B)
3. *Closure of the hernia ring* The hernia ring was stitched shut by continuous suture using a 2–0 barbed suture, and the pneumoperitoneum pressure could be appropriately reduced during the suture. (Fig. 2C)
4. *Mesh placement* A macroporous partially absorbable polypropylene mesh (ULTRAPRO Mesh; ETHICON Medical Devices Co., Ltd., Shanghai) was used in all three procedures. The four corners of the mesh were trimmed, the rolled mesh was inserted through the observation hole trocar, and the mesh was expanded to completely cover the hernia ring, with the edge exceeding the hernia ring at least by 5 cm (Fig. 2D). If the surgical field is clean, the placement of a negative pressure drainage tube is not necessary^5^, and no drains were placed in any of the three surgeries. The pneumoperitoneum was gradually relieved under direct vision, and the endoscope was withdrawn after making sure the mesh was not displaced and the coverage was flat, and the operation was completed.

## Results

All three operations were successfully completed. The intraoperative blood loss was about 10 ml, 7 ml and 5 ml, respectively. No urinary catheter or drainage tube was placed. The duration of the operations was 66 min, 105 min and 60 min, respectively. Following a successful general anesthesia recovery, three patients returned to the general surgical ward. The third patient required indwelling catheterization since she was unable to urinate by herself on the day of the surgery. The catheter was successfully withdrawn on the first day after surgery. All three patients resumed their normal diets and got out of bed on the first postoperative day without experiencing nausea, vomiting, abdominal pain, or distension. Postoperative pain was mild in all patients. On the second day following surgery, the visual analogue scale (VAS) for the first two patients was 1 and that for the third patient was 2. The first two patients were discharged on the second postoperative day, and the third patient was discharged on the third postoperative day. The postoperative follow-up time was 24 months, 23 months and 10 months, respectively. Considering reference and comparison with preoperative CT images, all three patients underwent abdominal CT examination at their first follow-up three months after surgery. Abdominal CTs did not reveal any recurrent hernias, and no effusion was found in the operative region. A follow-up CT image of the second patient is shown in Fig. 1G. Subsequent follow-up was conducted every six months, with a focus on physical examination.All three patients recovered well without any chronic pain. The patients’ perioperative data was summarized in Table 2.

**Table 2:** Patients’ perioperative data.

Patient	Operative time (mins)	Diameter of defect (cm)	VAS	LOS (days)	Follow-up (months)
1	66	3	1	2	24
2	105	3	1	2	23
3	60	3	2	3	10

*VAS* visual analog scale pain score under physical stress on postoperative day 2, *LOS* length of postoperative stay.

## Discussion

The upper lumbar triangle, often referred to the Grynfeltt-Lesshaft triangle, is an inverted triangle with the upper boundary being the lower edge of the 12th rib, the inner boundary being the lateral edge of the quadratus lumborum, the outer boundary being the posterior edge of the internal oblique muscle, and the transversus abdominis aponeurosis at the bottom^5^. The lower lumbar triangle, also known as the Petit triangle, is bordered by the external oblique muscle laterally, the iliac crest at the base, the latissimus dorsi muscle medially, and the floor is formed by the deep lumbar fascia^12^. Due to the fact that the upper lumbar triangle space is larger than the lower lumbar triangle space and does not have complete muscle coverage, while the lower lumbar triangle bottom is protected by the internal oblique muscle, the incidence of upper lumbar hernia is higher^16^. In our study, the two primary lumbar hernias were both upper lumbar hernias, and the additional patient had a secondary lumbar hernia.

Although a lumbar hernia instance was documented as far back as the seventeenth century^17^, its relatively rare incidence has limited the development of surgical repair techniques. Currently, there is no unified standard for the treatment of lumbar hernia^18^. In general, the lumbar hernia may be accompanied by low back pain and other symptoms, and may also cause serious complications, such as incarceration and strangulation^11^. Moreover, the operation is safe and has no serious complications. Therefore, the majority of academics advocate that surgical treatment should be performed as soon as possible if there is no obvious surgical contraindication^19^. At present, the most commonly used surgical techniques can be divided into three categories: (1) traditional open Sublay; (2) intraperitoneal onlay mesh (IPOM); and (3) transabdominal preperitoneal repair (TAPP) and transabdominal partially extraperitoneal repair (TAPE). As compared to the latter two types of procedures, open surgery has the longest history and the lowest requirements for the surgeon. However, because the scope of the hernia ring defect cannot be properly assessed through palpation, it usually requires a large incision, which results in more trauma for the patient and a longer hospital stay^10,20^. The surrounding bony structure may become one of the obstacles to surgical exposure and adequate mesh overlap and fixation^21^ In addition, the subcostal nerve can be cut or injured by a lateral lumbodorsal fascial incision, which may lead to muscle weakness and subsequent larger lumbar hernia^21^. Sometimes open surgery even involves the use of complex flaps^7^, which increases the risk of mesh infection or hernia recurrence. The latter two types of surgical procedures are more commonly used in clinical practice because they have a large operating space, can accurately locate the hernia sac and evaluate the defect, and cause less trauma^22,23^^.^ However, they also have some disadvantages, such as the requirement for abdominal cavity entry to incise the lateral peritoneum and the partial dissociation of the ascending colon and even the kidney, which increases the risk of visceral injury. At the same time, expensive anti-adhesion mesh and invasive nail gun fixing are required^11,12^.

We have recently successfully performed retroperitoneal totally endoscopic prosthetic repair (R-TEP) for 3 patients with lumbar hernias. Based on our own surgical experience and previous literature reports^1,14,15^, we believe that R-TEP is a more reasonable surgical procedure. Despite the smaller surgical space and more difficulty, it avoids the shortcomings of the aforementioned surgical techniques. Its advantages include the following: (1) laparoscopic three-hole operation, less trauma and quick recovery; (2) walking on the natural anatomical level to avoid additional trauma; (3) the operation does not enter the abdominal cavity without mobilization of intraperitoneal structures; (4) only use ordinary polypropylene mesh, greatly reduce medical costs; and (5) the mesh makes no contact with the abdominal cavity, reducing the risk caused by foreign bodies in the abdominal cavity.

When performing R-TEP, a few operative points need to be taken into consideration. First, the retroperitoneal region is the junction and fusion area of endoderm, mesoderm and ectoderm^24^. Understanding the anatomy will help us to separate at the correct level, avoid damaging the diaphragm, and prevent chronic postoperative pain caused by nerve damage. The principal nerves that run in front of the upper lumbar triangle are the subcostal nerve, iliohypogastric nerve, and ilioinguinal nerve, which perforate from the outer edge of the psoas major muscle and then run in front of the quadratus lumborum muscle (Fig. 2B). Second, the separated retroperitoneal area should be slightly larger than the mesh; otherwise, the mesh could be easily displaced or difficultly spread. Thirdly, if the hernia sac is broken during the separation process, a separation forcep can be used to clamp the tear. After that, a figure-of-eight suture is applied below the tear using a 4–0 absorbable suture. To close the tear, tighten the suture, loosen the separation forcep, and tie a knot. High proficiency in laparoscopic single-hand suture is necessary for this technique. You can also use a fisherman's knot to ligate the tear. When hospital conditions permit, an automatic ligation device can be positioned beneath the tear and tightened to seal it. This method is more convenient. Fourth, interrupted sutures from outside the body through the abdominal wall using a hooked needle can be used to close the hernia ring if the tension is high or the surgeon's laparoscopic suture ability is inadequate. In order to avoid a local dead cavity forming after the hernia back and subsequent postoperative effusion and even infection, the loose Scarpa's fascia outside the hernia ring defect should be attached during suture. Fifth, the edge of the mesh should be as flat as possible to avoid curling to prevent possible foreign body sensations following surgery.

The selection and fixation of mesh is a topic worth discussing. We have chosen a macroporous partially absorbable mesh. The scars formed by themacroporous material are elastic and can reduce the pain caused by the compression of nerves by plate shaped scars^25^. Partially absorbable material ensure strength in the early postoperative period while remaining less, resulting in milder inflammatory reaction and less foreign body feeling and pain^26,27^. None of the three patients mentioned above had a hernia ring larger than 3 cm in diameter, and none had an intraoperative mesh fixed. All three patients had great postoperative follow-up outcomes with no lumbar hernia recurrence on repeat abdominal CT scans. Preliminarily, it can be shown that as long as the mesh area is sufficiently large and smooth, intra-abdominal pressure can often fix the mesh properly when the hernia ring defect is not large^8,9^. We currently have no experience in the fixation of mesh when the hernia ring diameter exceeds 3 cm. Li^1^ suggested using chemical glue to fix the mesh. He believed that a traumatic fixation device was not necessary because of sufficient overlapping. However, we have a different point of view. When the hernia ring defect is large, the mesh is needed to provide not only localized membranous reinforcement, but also to address the issue of muscle stress in various directions during postoperative trunk movement. The effect of stress and the inevitable contracture of the mesh may lead to the displacement of the mesh. At this point, it is crucial that the mesh, in particular the edge portion, is effectively fixed in the stress direction. Fixation techniques include suturing, nail fixation, etc. Fixation at the hernia ring, iliac crest, anterior border of the psoas major, and the 11th rib respectively^28^. During fixation, nerves damage should be avoided to prevent postoperative chronic pain, and attention should be paid to avoid damage to nearby tissues and organs. For instance, the upper boundary of the upper lumbar triangle is the 12th rib, fixation nearby should avoid injury to the pleura and anterior renal tissue.

**Figure 3:**
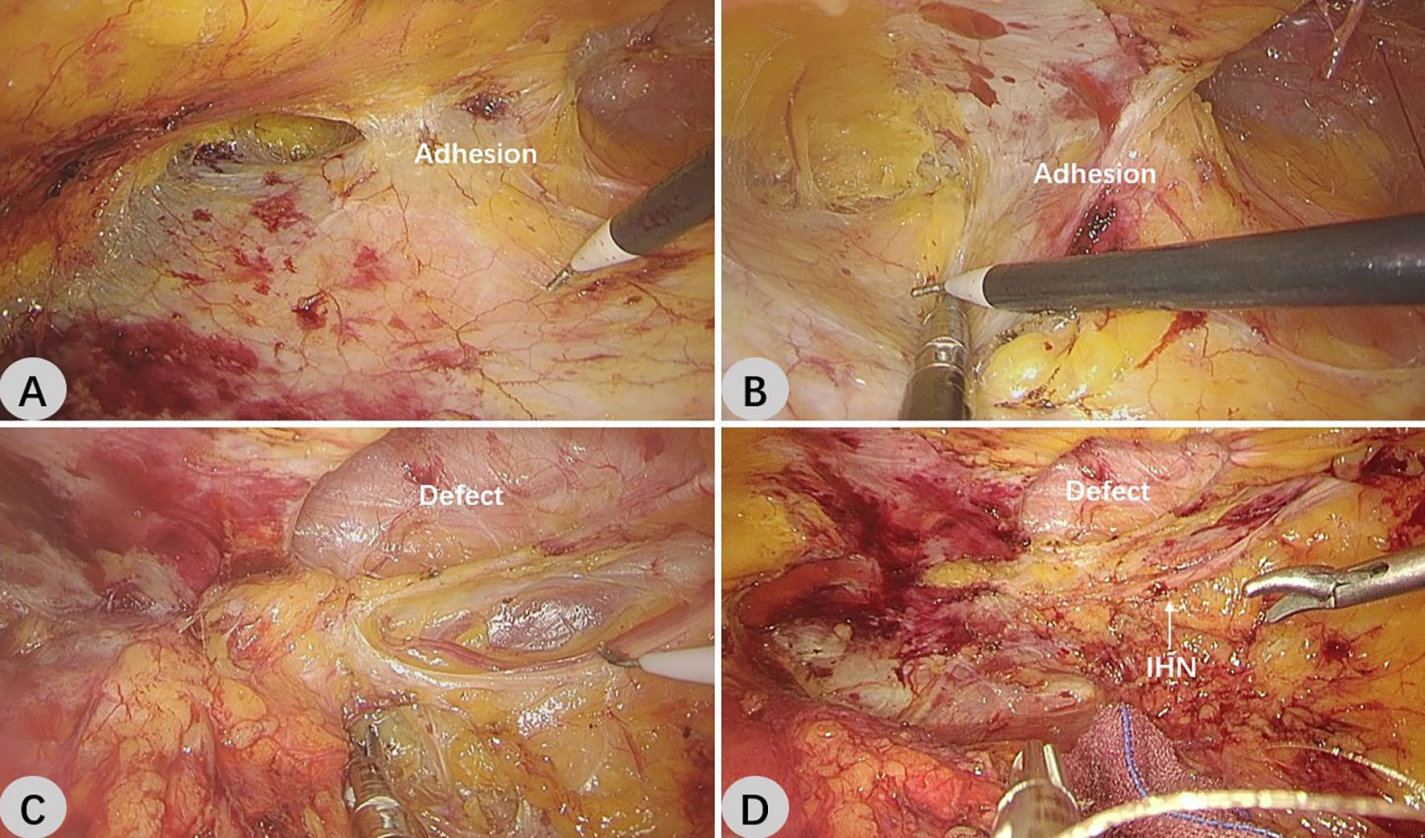
Intraoperative images of R-TEP in the treatment of secondary lumbar hernia (**A**,**B**) Cicatricial adhesions in the retroperitoneal space of the secondary lumbar hernia. (**C,D**) The surgical area of the secondary lumbar hernia is disordered and the anatomical level is unclear. Note the protecting of the nerves.

Some academics believe that the surgical treatment of secondary lumbar hernia benefits greatly from the laparoscopic technique. On one hand, a skin incision in laparoscopic surgery is far from the scar tissue; on the other hand, open surgery involves a lot of dissection, which will increase the risk of postoperative complications^9^. Previous reports on R-TEP have largely been for primary lumbar hernias. We successfully performed R-TEP for a patient with a secondary lumbar hernia. Following the resection of the left renal cyst, this patient developed a trocar hernia in the left upper lumbar triangle. There were several experiences to be shared during the R-TEP procedure. First, the extraperitoneal space of the secondary lumbar hernia often has cicatricial adhesions, especially in the vicinity of the hernia sac, making it difficult to establish an effective surgical space (Fig. 3A, B). Second, the surgical area of the secondary lumbar hernia is disordered and the anatomical level is unclear, making it easy to damage the transverse abdominal fascia and also difficult to protect the iliohypogastric nerve and ilioinguinal nerve (Fig. 3C, D). In order to make it easier to find the right level, the operation should adhere to the "easy first and difficult later" approach by first attempting to separate the surrounding loose space and then separating the dense adhesion area, primarily via blunt separation. A constant and sufficient local tension should be provided when utilizing the electric hook for sharp separation. The tissue should not be forcibly cut under low tension, otherwise, it will not only destroy the layer, but also easily break the hernia sac or peritonea. We believe that R-TEP is feasible for the treatment of secondary lumbar hernia through the improvement of technique and accumulation of experience.

## Conclusion

In light of the relevant literature that has been published so far and our own experiences, R-TEP for lumbar hernia is safe and feasible under the premise of proficient anatomy and certain laparoscopic technique. However, the technical details of the surgery require further refinement and standardization. In view of the small number of cases studied worldwide, particularly secondary lumbar hernias with only one case reported in our study and one case reported by Meink^14^, and the short follow-up period, a larger sample of data and long-term follow-up observation are needed to further explore the clinical value of R-TEP for the treatment of lumbar hernia.

## Data Availability

The datasets used and/or analyzed during the current study available from the corresponding author on reasonable request.
